# NADPH oxidase 4 modulates hepatic responses to lipopolysaccharide mediated by Toll-like receptor-4

**DOI:** 10.1038/s41598-017-14574-8

**Published:** 2017-10-30

**Authors:** Anand Singh, Bhargav Koduru, Cameron Carlisle, Hasina Akhter, Rui-Ming Liu, Katrin Schroder, Ralf P. Brandes, David M. Ojcius

**Affiliations:** 1 Health Sciences Research Institute, University of California, Merced, CA USA; 2 School of Medicine, University of Alabama at Birmingham, Birmingham, AL USA; 3 Institute for Cardiovascular Physiology, Goethe Universität, Frankfurt am Main, Germany; 4 University of the Pacific, Arthur Dugoni School of Dentistry, San Francisco, CA USA

## Abstract

Chronic inflammation plays a key role in development of many liver diseases. Stimulation of Toll-like receptor 4 (TLR4) by bacterial lipopolysaccharide (LPS) initiates inflammation and promotes development of hepatocellular carcinoma and other liver diseases. NADPH oxidases contribute to LPS-induced reactive oxygen species (ROS) production and modulate TLR responses, but whether these enzymes function in TLR4 responses of hepatocytes is unknown. In the present work, we examined the role of NADPH oxidase 4 (Nox4) in LPS-induced TLR4 responses in human hepatoma cells and wildtype and Nox4-deficient mice. We found that LPS increased expression of Nox4, TNF-α, and proliferating cell nuclear antigen (PCNA). Nox4 silencing suppressed LPS-induced TNF-α and PCNA increases in human cells. The LPS-induced TNF-α increases were MyD88-dependent, and were attenuated in primary hepatocytes isolated from Nox4-deficient mice. We found that Nox4 mediated LPS-TLR4 signaling in hepatocytes via NF-ĸB and AP-1 pathways. Moreover, the effect of Nox4 depletion was time-dependent; following six weeks of repeated LPS stimulation *in vivo*, hepatic TNF-α and PCNA responses subsided in Nox4-deficient mice compared with wildtype mice. Therefore, our data suggest that Nox4 mediates LPS-TLR4 signaling in human hepatoma cells and murine hepatocytes and may contribute to the ability of LPS to stimulate liver pathology.

## Introduction

Hepatic inflammation is an important contributing factor in chronic liver diseases. Chronic liver injury and cirrhosis caused by persistent inflammation precede approximately 80% of hepatocellular carcinoma (HCCs) cases, the third leading cause of cancer mortality worldwide^1,2^. The liver is constantly exposed to gut-derived microbial products, including lipopolysaccharides (LPS), a cell-wall component of Gram-negative bacteria, that contributes to inflammation. Increased levels of LPS in portal and systemic circulation is a common finding in patients with chronic liver disease^3–5^. The increased LPS levels are associated with functional impairment of the gut barrier and increased translocation of intestinal bacteria^6^.

Toll-like receptors (TLRs) comprise a family of receptors that recognize diverse ligands, including pathogen-associated molecular patterns (PAMPs)^7,8^. TLRs play critical roles in innate immunity as well as subsequent induction of adaptive immune responses against microbial pathogens. For example, binding of LPS to TLR4 triggers a signaling pathway, leading to activation of transcription factors, such as activator protein-1 (AP-1) and nuclear factor κB (NF-κB), and the subsequent production of proinflammatory cytokines. The TLR4 responses are mediated by myeloid differentiation primary response gene 88 (MyD88), an adaptor protein^9,10^. Evidence indicates that hepatocytes, Kupffer cells, and hepatic stellate cells of the liver each express TLR4 and may thus play important roles in hepatic inflammation and fibrosis triggered by LPS^11,12^. Recent studies indicate that the activation of TLR4 by LPS derived from intestinal microbiota contributes to inflammation and promotion of liver injury-mediated HCC, while removal of intestinal microbiota can decrease the incidence of HCC^4,13,14^.

The literature suggests that LPS induced TLR4 signaling leads to enhanced production of reactive oxygen species (ROS), which in turn activate downstream signaling cascades^15–18^. For example, NADPH oxidase 4 (Nox4) has been identified as a source of the LPS-induced ROS and proinflammatory responses in human HEK293T cells, U937 monocytic cells, human aortic endothelial cells, and human renal mesanglial cells^18–21^. The Nox family consists of five Nox proteins (Nox1-5) and two dual oxidases (Duox1 and Duox2) that catalyze the transfer of electrons from NADPH to oxygen to produce superoxide and hydrogen peroxide^22^. Studies indicate that the liver expresses Nox family proteins and that hepatic Nox proteins contribute to hepatic fibrosis stimulated by bile duct ligation^23–26^. However, the role of Nox4 in the LPS induced hepatic responses and particularly, in hepatocytes, is unknown.

The goal of this study, therefore, is to evaluate the role of Nox4 in the LPS induced responses in a well-characterized human hepatoma cell line, primary hepatocytes isolated from control wildtype (Nox4^+/+^) and Nox4-deficient (Nox4^−/−^) mice, and in these animals *in vivo*. We present evidence that Nox4 mediates LPS-induced TLR4 signaling in human hepatoma cells and murine hepatocytes as well as liver and plasma in whole mice *in vivo*.

## Materials and Methods

### Reagents and antibodies

Dulbecco’s modified Eagle’s medium (DMEM), fetal bovine serum (FBS), and 0.25% trypsin-EDTA, penicillin and streptomycin (PenStrep) were purchased from Invitrogen (Carlsbad, CA, USA). 5-(and-6)-chloromethyl-2,7-dichlorodihydrofluoresceindiacetate, acetyl ester (CM-H2DCFDA) was obtained from Molecular Probe (Invitrogen). LPS (*Escherichia coli* 0127:B8) was purchased from Sigma-Aldrich (St. Louis, MO). The following primary antibodies were used in this study: rabbit monoclonal anti-Nox4,rabbit polyclonal anti-TNFα, rabbit polyclonal anti-TGFβ1 (Abcam, Cambridge, MA, USA); rabbit polyclonal anti-Nox4 (Millipore, Billerica, MA, USA); rabbit polyclonal anti-NFĸB p65, rabbit polyclonal anti-PCNA, goat polyclonal anti-GAPDH, goat polyclonal anti-actin (Santa Cruz Biotechnology, CA, USA); rabbit monoclonal anti-ERK1/2, rabbit monoclonal anti-MyD88 (Cell Signaling Technology, MA, USA); mouse monoclonal Anti-ERK1/2 (pT202/pY204) (BD Biosciences, USA) and rabbit polyclonal anti-NFĸB p65(Ser536) (Bioss Antibodies, USA).

### Cell culture

Huh7 human hepatoma cells (Japanese Collection of Research Bioresources Cell Bank, Japan) were cultured in DMEM supplemented with 10% fetal bovine serum, 100 U/ml penicillin, 100 μg/ml streptomycin sulfate, and 2.0 mM glutamine. Cells were maintained in a humidified incubator with 5% CO_2_ at 37 °C and passaged using 0.25% trypsin-EDTA. For *in vitro* LPS treatments, cells were incubated with 1 μg/ml LPS for up to 72 hours, as indicated in Results.

### Animals

C57BL/6 J wild type mice and Nox4 knockout (Nox4^−/−^) with same background generated by coauthors as described previously^27^, were used for this study, Nox4 knockout animals were rederived at the University of California, Merced animal facility. The study was approved by the Institutional Review Boards at Lawrence Livermore National Laboratory and University of California, Merced. All the experiments described here were performed in accordance with relevant guidelines and regulations. Wild type and Nox4 knockout male mice (8–12 weeks old) were injected with saline (0.9% NaCl) only or LPS (1 mg/kg body weight) dissolved in saline *i*.*p*. once per week for up to six weeks unless indicated otherwise in Results.

### Primary hepatocytes

Primary hepatocytes were isolated from control wild type and Nox4 knockout mice (10–12 weeks old), by sequential perfusion of liver with EDTA and collagenase as previously described^28,29^. Then, 1 × 10^6^ cells/2 ml/well were seeded in six wells collagen-coated tissue culture plates. After 24 hrs, cells were stimulated with PBS or 1 μg/ml LPS for 3 hrs and harvested for analysis. Albumin staining was done to verify hepatocyte isolation.

### Determination of intracellular H_2_O_2_ production

Intracellular production of H_2_O_2_ was measured in Huh-7 cells after 3 hours of LPS (1 μg/ml) stimulation. Cells were washed twice with HBSS and incubated in HBSS containing 5 μM CM-H2DCFDA for 15 min in the dark at 37 °C, as described previously^20,30^. Intracellular H_2_O_2_ levels were measured with a laser-scanning confocal microscope (Eclipse Ti C1, Nikon). Where indicated, cells were pretreated with either control/Nox4 siRNA for 24 hours or diphenyliodonium for 30 min before LPS stimulation and ROS measurement. All experiments were repeated at least three times.

### Immunofluorescence Staining

Cells were fixed with 3.5% formaldehyde for 10 minutes and permeabilized with phosphate-buffered saline containing 1% (wt/vol) bovine serum albumin, 0.02% (wt/vol) saponin and 0.05% (wt/vol) sodium azide. Subsequently, samples were incubated with primary and then fluorophore-conjugated secondary antibodies, and imaged by using confocal laser scanning microscopy^31^. DAPI (4′,6-diamidino-2-phenylindole) was used for counter staining. Images were quantified by ImageJ software available at http://rsbweb.nih.gov/ij/.

### Small Interfering RNA (siRNA) transfection

Huh7 cells were transfected with Nox4 or non-targeting control siRNA (50 nM; smartpool siRNAs Dharmacon), using Lipofectamine RNAiMax (Invitrogen,USA), according to the manufacturer’s protocol.

### Determination of messenger RNA (mRNA) levels

Total intracellular RNA was extracted using Trizol (Invitrogen), and the concentration was determined using NanoDrop 2000 spectrophotometer (Thermo Fisher Scientific, USA). TNF-α, TGF-β1, Nox4, TLR4, PCNA, MyD88, and GAPDH mRNA levels were quantified by quantitative real time reverse transcriptase-polymerase chain reaction (qRT-PCR), using Power SYBR Green PCR Master Mix (Applied Biosystems) or EXPRESS One-Step SYBR® GreenER™ Universal (Invitrogen), using ABI-7300 Real-Time PCR (Applied Biosystems, USA). Glyceraldehyde 3-phosphate dehydrogenase (GAPDH) mRNA level was also determined as control. Primer sequences used for TNF-α, TGF-β1, Nox4, TLR4, PCNA, MyD88 and GAPDH are listed in Supplementary Table I.

### Western blot analysis

Total cell lysates were prepared using radioimmunoprecipitation assay buffer (RIPA) or 2x Laemmli buffer supplemented with proteinase inhibitor cocktail (Sigma, USA) and phosphatase inhibitor cocktail (Sigma,USA). Liver samples were lysed and sonicated in T-PER (Thermo Fisher Scientific, Rockford, USA) per manufacturer’s protocol. Protein concentrations were determined using abicinchoninic acid (BCA) protein assay kit (Thermo Fisher Scientific, Rockford, USA). Protein levels were analyzed by Western blot, as previously described^31,32^. β-Actin or GAPDH levels were also determined as controls. Protein bands were detected by chemiluminescence (Kodak Digital Science Image Station) or infrared fluorescence (Odyssey Image station and Image Studio, LI-COR Biosciences, Lincoln, NE, USA).

### Luciferase assay

Cells were transfected with control or Nox4 siRNA. After 24 hrs, co-transfected with an NF-ĸB- or AP1 luciferase reporter plasmid and β-galactosidase (βgal) construct using Lipofectamine LTX with Plus reagent per manufacturer’s protocol (Invitrogen). After 24 hrs, cells were replenished with fresh media supplemented with LPS (1 µg/ml). After 6 hrs of LPS stimulation, luciferase activity was determined, using Luciferase Reporter Assay System (Promega Corporation, Madison, WI) according to manufacturer’s protocol. Data were normalized by βgal activity, determined using β-Galactosidase Enzyme Assay system (Promega Corporation, Madison, WI).

### Cell proliferation assay

Cell proliferation was monitored, using CyQUANT® Direct Cell Proliferation Assay Kit (Invitrogen, USA), according to manufacturer’s protocol.

### Nox activity assay

Nox activity was determined from cells, using cytochrome c reduction assay, as described^31^. Liver samples were sonicated in an intracellular-like buffer (see ref.^31^ for composition), supplemented with protease inhibitor cocktail (Sigma-Aldrich), and filtered through 0.2 µm filter (Millipore). Then, Nox activity was determined in intracellular-like buffer, containing 150 µM cytochrome c,100 µM NADPH, 100 µM ATP, 100 µM GTP, and 100 µg protein per reaction^33^, as described above. Protein concentration was determined, using aBCA kit (ThermoScientific).

### Enzyme linked immunosorbent assay (ELISA)

Tumor necrosis factor alpha levels in the plasma samples from mice were determined, using TNF-α DuoSet^R^ ELISA development system (R&D system), asper manufacturer’s protocols.

### Statistical analysis

Data are presented as mean ± standard error of the mean (SEM). All experiments were performed in duplicates or triplicates and repeated at least three times. Data were analyzed using Student’s t test or one-way analysis of variance, using GraphPad Prism version 5.00 (GraphPad Software, La Jolla California USA). A p value ≤ 0.05 was considered statistically significant.

## Results

### LPS increased Nox4 levels in Huh7 human hepatoma cells

**Figure 1:**
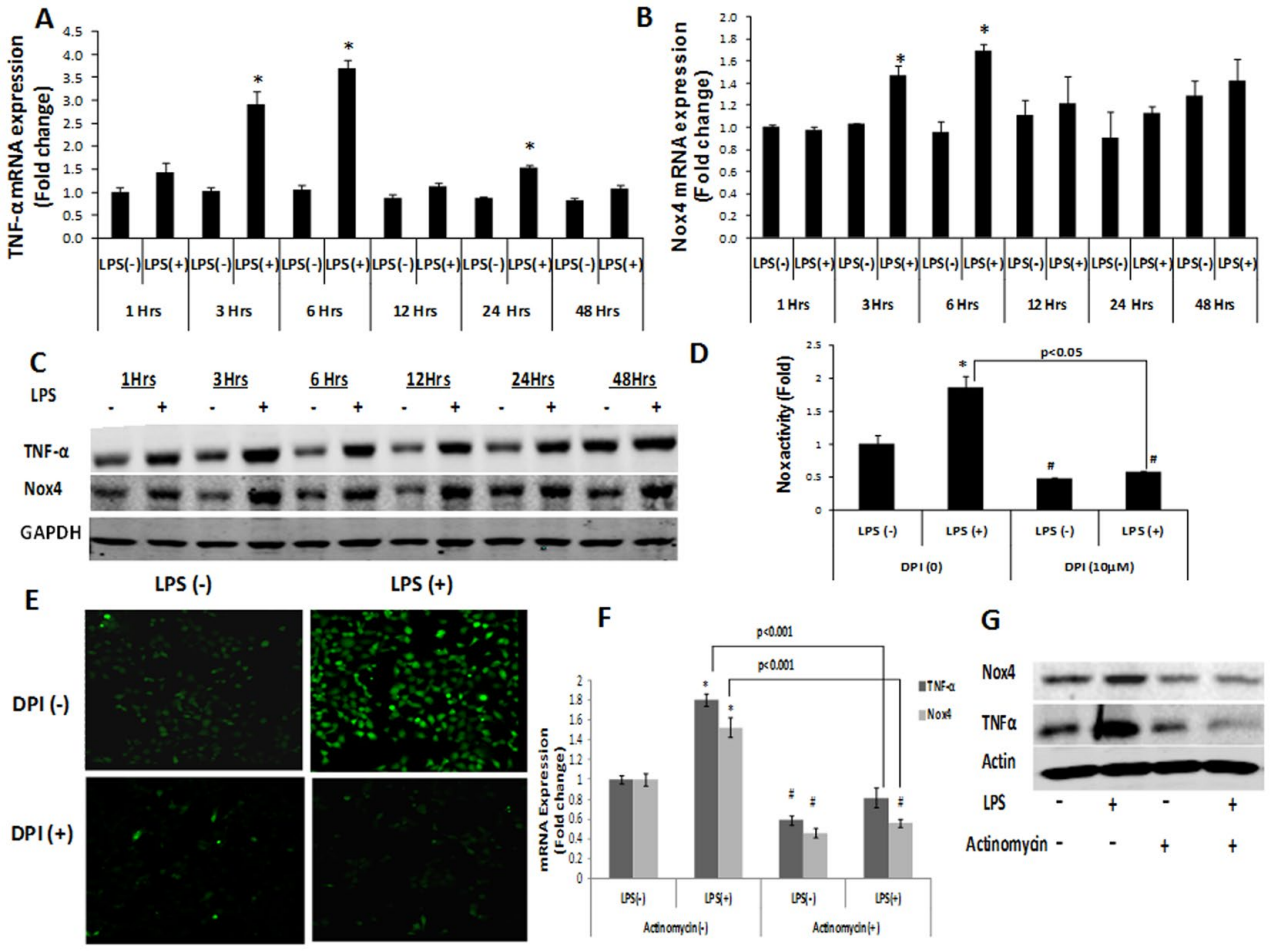
LPS stimulation increased TNF-α and Nox4 expression. Huh7 cells were either treated with LPS (1 µg/ml) or PBS for up to 48 hrs, and analyzed for TNF-α (**A**) and Nox4 (**B**) mRNA expression by qRT-PCR. Data was normalized with GAPDH mRNA and calculated by ΔΔCt method and expressed as fold increase from the control. (**C**) TNF-α and Nox4 protein levels in Huh7 cells after LPS treatment were determined by western blotting. (**D**) Nox enzyme activities were determined in Huh7 cells either untreated or pretreated with DPI (10 µM) for 30 minutes before 6 hrs of LPS stimulation by monitoring NADPH-dependent and SOD-inhibited reduction of cytochrome c, as described in materials and methods. ^#^ indicates statistically significant difference from DPI (0) LPS (−) (p < 0.05). (**E**) Huh7 cells either untreated or pretreated with DPI (10 µM) for 30 minutes before 3 hrs of LPS stimulation. Then, cells were labeled with 5 µM CM-H2DCFDA for 15 min and intracellular generation of H_2_O_2 _was monitored by confocal microscopic analysis of DCF fluorescence. (**F**,**G**) Nox4 and TNF-α mRNA and protein levels in Huh7 cells either untreated or treated with Actinomycin D (1 µM) for 30 minutes before 3 hrs of LPS stimulation. ^#^ indicates statistically significant difference from control LPS (−) (p < 0.05). * indicates statistically significant difference from the corresponding controls (p < 0.05). Lines with p values also indicate statistically significance (p < 0.05) between the groups.

To determine whether Nox4 is involved in the LPS-induced responses in hepatocytes, Huh7 cells were stimulated with LPS for up to 48 hours and analyzed for tumor necrosis factor alpha (TNF-α) and Nox4 mRNA levels by qRT-PCR. We found that LPS increased TNF-α as well as Nox4 mRNA levels in Huh7 cells (Fig. 1A and B). Similarly, TNF-α and Nox4 protein levels were elevated after the LPS treatment ((Fig. 1C and Supplementary Figs S1 & S2). These results suggest that LPS increases TNF-α and Nox4 levels in hepatocytes.

Furthermore, Nox activity, quantified using permeabilized Huh7 cells, showed elevated Nox enzyme activity upon LPS treatment that responded to suppression by diphenyleneiodonium (DPI), a commonly used inhibitor of Nox proteins (Fig. 1D). The generation of intracellular ROS (H_2_O_2_) in Huh7 cells was measured by oxidation of H2DCFDA to DCF using confocal microscopy. LPS treatment increased the generation of ROS as revealed by an increase in DCF fluorescence (Fig. 1E and Supplementary Fig. S3).

TNF-α and Nox4 mRNA levels were next analyzed in Huh7 cells pretreated with actinomycin D^34^, before the LPS stimulation. The LPS-induced increases in TNF-α and Nox4 mRNA and protein were decreased with actinomycin D treatment (Fig. 1F and G), indicating that the LPS-induced increases in the TNF-α and Nox4 levels is transcription-dependent in these cells.

### Upregulation of proliferating cell nuclear antigen (PCNA) by LPS

**Figure 2:**
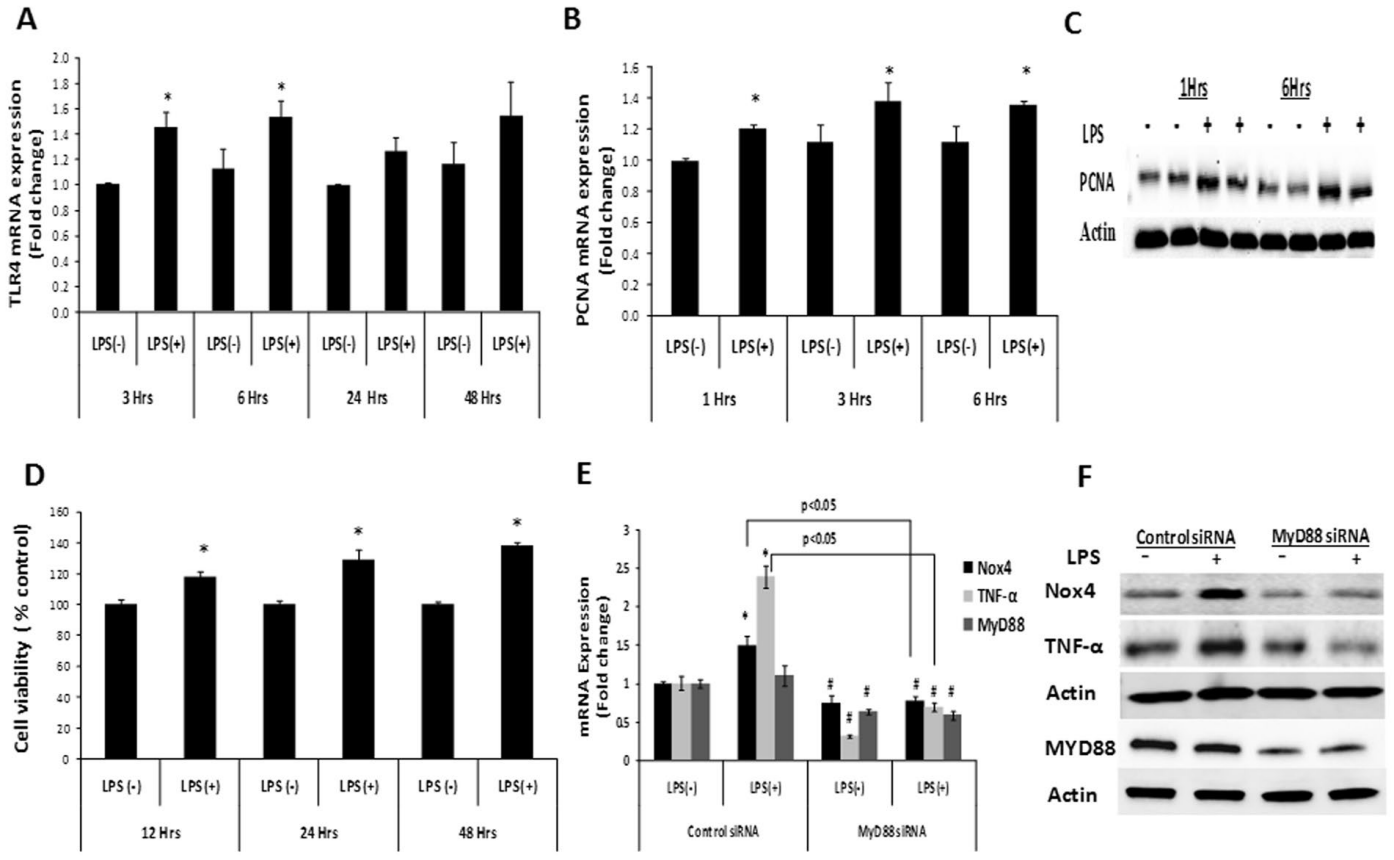
LPS increased PCNA expression and LPS-TLR4 signaling in hepatoma cells is MyD88 dependent. Huh7 cells were either treated with LPS or PBS for indicated time points and analyzed for TLR4 (**A**) and PCNA (**B**) mRNA expression by qRT-PCR. (**C**) PCNA protein levels were measured by western blotting in Huh7 cells treated either LPS or PBS at indicated time points. Actin was used as a control for protein loading. (**D**) Huh7 cells were either treated with LPS or PBS for indicated time points and viable cells were measured by cell proliferation assay. (**E**) Huh7 cells were transfected with control or MyD88 siRNA for 48 hrs, and treated either with LPS or PBS for an additional 3 hrs. Then, samples were analyzed for Nox4, TNF-α and MyD88 mRNA levels by qRT-PCR. (**F**) Nox4, TNF-α and MyD88 protein levels were measured by western blotting and actin was used as a loading control. ^#^ indicates statistically significant difference from control siRNA LPS (−) (p < 0.05). * indicates statistically significant difference from the corresponding controls (p < 0.05). Lines with p values also indicate statistically significance (p < 0.05) between the groups.

Recent studies suggest that activation of TLR4 with ligand LPS may promote hepatocellular carcinoma (HCC) by increasing proliferative signals in resident liver cells and the liver^4,13,14^. Therefore, the expression of TLR4 and a proliferation marker, PCNA, were examined following LPS treatment. The results showed that LPS increased TLR4 expression in Huh7 cells (Fig. 2A). We also found a significant increase in PCNA mRNA levels with LPS stimulation (Fig. 2B). Western blot analysis confirmed increased PCNA protein levels after LPS treatment (Fig. 2C and Supplementary Fig. S4). Next, Huh7 cell proliferation was measured upon LPS stimulation; a proliferation assay showed a significant increase in cell proliferation after LPS treatment (Fig. 2D). These data suggest that LPS-TLR4 signaling may affect hepatocyte proliferation.

### LPS induced TNF-α and Nox4 expression *via* the MyD88-dependent pathway

TLR4 signaling involves MyD88-dependent and MyD88-independent pathways^9,10^. To test whether the LPS activated Nox4 in Huh7 cells through MyD88, Huh7 cells were transfected with MyD88 siRNA or control non-targeting siRNA, followed by LPS stimulation, and analyzed for MyD88, TNF-α, andNox4 mRNA levels. The results showed that LPS increased Nox4 and TNF-α mRNA levels in the control siRNA treated cells without significant alteration of MyD88 mRNA levels (Fig. 2E). Depletion of MyD88 with MyD88 siRNA suppressed the LPS-induced increases in TNF-α and Nox4 mRNA levels (Fig. 2E). Western blot analysis confirmed increased Nox4 and TNF-α protein levels in control siRNA-treated cells after LPS treatment, while MyD88 depletion decreased LPS-induced Nox4 and TNF-α protein levels (Fig. 2F). These data suggest that the elevation of TNF-α and Nox4 by LPS in human hepatoma cells is mediated by MyD88.

### Nox4 silencing and DPI suppress LPS-induced TNF-α and PCNA elevation

**Figure 3:**
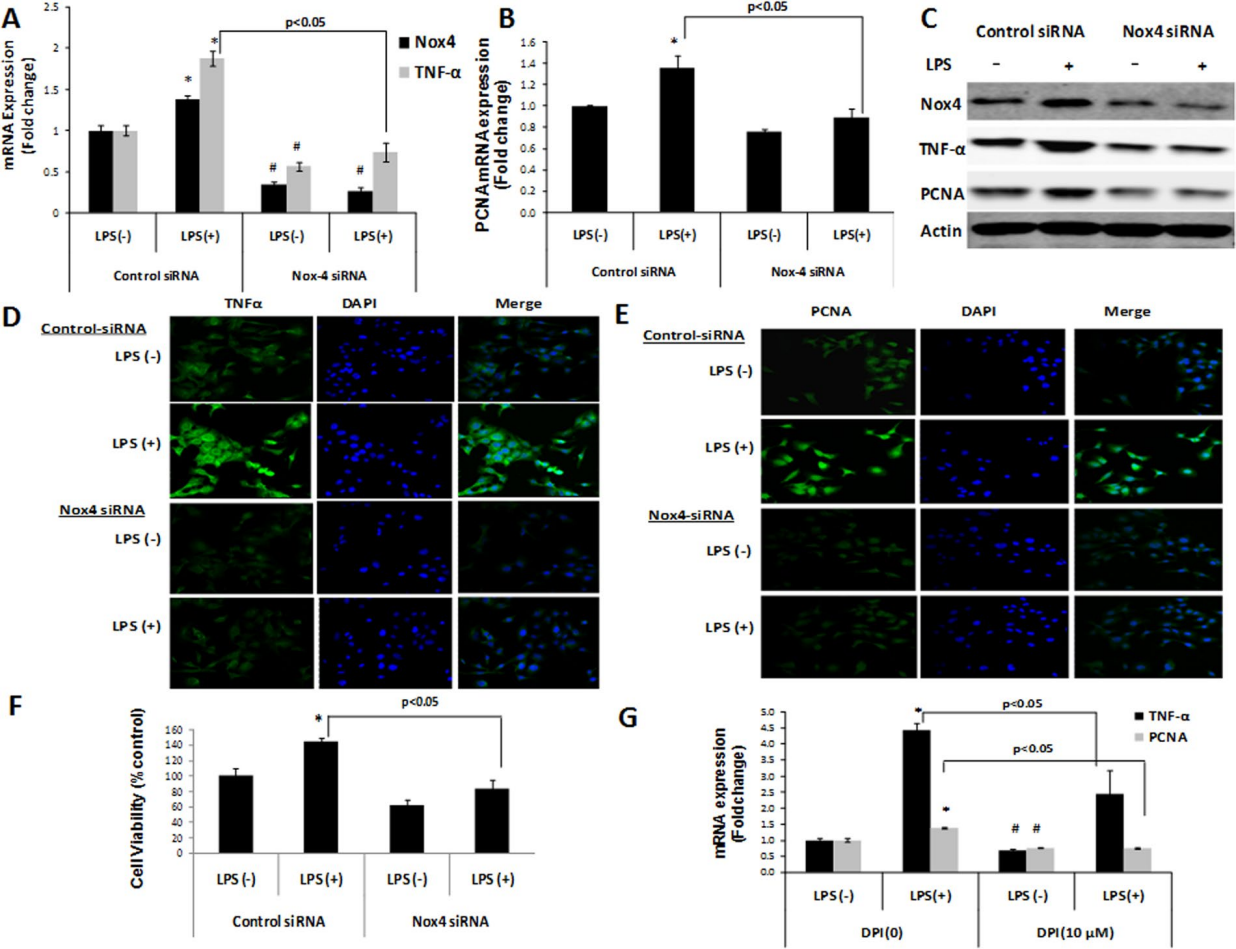
Nox4 siRNA and DPI suppressed LPS induced TNF-α and PCNA elevation. Nox4, TNF-α (**A**) and PCNA (**B**) mRNA levels were analyzed by qRT-PCR in Huh7 cells transfected with control or Nox4 siRNA for 48 hrs, and either treated with LPS or PBS for an additional 3 hrs. (**C**) Nox4, TNF-α and PCNA protein levels were determined by western blotting in Huh7 cells treated with controlor Nox4 siRNA for 48 hrs, and either treated with LPS or PBS for an additional 3 hrs. Actin was used as a control for protein loading. (**D**,**E**) Huh7 cells treated with controland Nox4 siRNA for 48 hrs,and either untreated or treated with LPS for an additional 3 hrs were analyzed for the cellular level of TNF-α (**D**), and PCNA (**E**) protein by confocal microscopy. Confocal microscopic fields are shown in (**D**,**E**), a representative picture of three independent experiments. (**F**) Huh7 cells transfected with control and Nox4 siRNA for 24 hrs, and either treated with LPS or PBS for an additional 24 hrs. Then, cell numbers were measured by cell proliferation assay. (**G**)TNF-α and PCNA mRNA levels were analyzed by qRT-PCR in Huh7 cells either untreated or pretreated with DPI (10 µM) for 30 minutes before 3 hrs of LPS stimulation. * indicates statistically significant difference from the corresponding controls (p < 0.05). ^#^ indicates statistically significant difference from control siRNA LPS (−) or DPI (0) LPS (−) (p < 0.05). Lines with p values also indicate statistically significance (p < 0.05) between the groups.

To investigate the potential role of Nox4 in the MyD88-dependent TLR4 signaling pathway in hepatocytes, Nox4-depleted Huh7 cells were stimulated with LPS. Nox4 siRNA decreased Nox4 mRNA and protein levels (Fig. 3A and C) and baseline as well as LPS-stimulated TNF-α mRNA levels in Huh7 cells (Fig. 3A). Western blot and immunofluorescence staining also revealed decreases in TNF-α protein levels in Nox4 siRNA versus control siRNA group (Fig. 3C and D). Nox4 siRNA decreased baseline as well as LPS induced ROS levels in Huh7 cells compared to control siRNA treated cells (Fig. Supplementary Fig. S5). Nox4 siRNA likewise attenuated the LPS-induced PCNA elevation (Fig. 3B and E) and markedly decreased proliferation of Huh7 cells upon LPS stimulation (Fig. 3E). Furthermore, pretreating Huh7 cells with DPI attenuated LPS-stimulated TNF-α and PCNA mRNA levels (Fig. 3G). These data indicate that Nox4 may affect inflammation and cell proliferation by modulating LPS-TLR4 responses in hepatoma cells.

### Deletion of the Nox4 gene attenuates LPS-induced TNF-α expression in primary hepatocytes in mice

**Figure 4:**
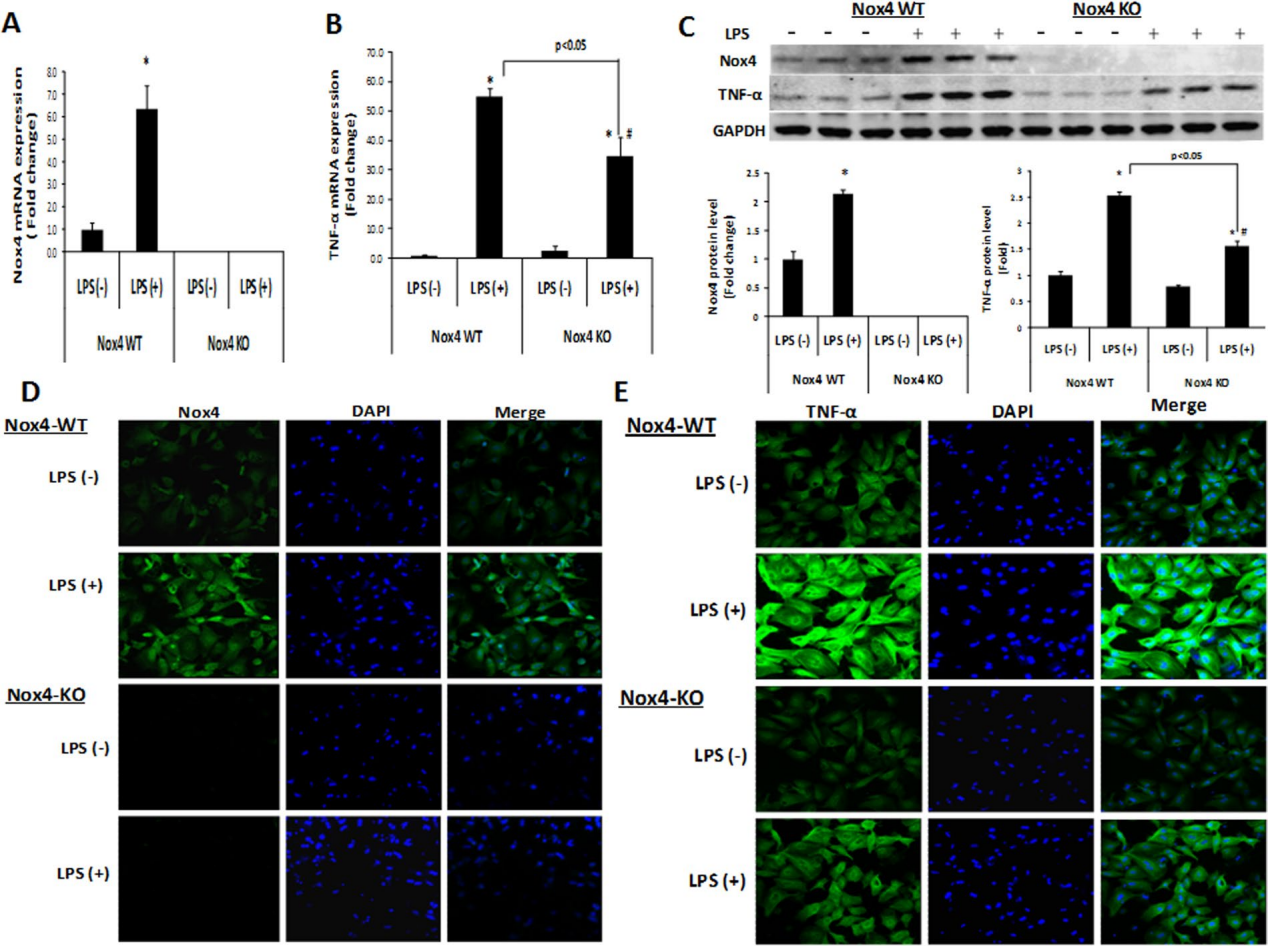
LPS induced TNF-α levels attenuated in primary hepatocytes of Nox4 KO mice. Primary hepatocytes isolated from Nox4 WT (n = 3) and Nox4 KO (n = 3) mice were either treated with PBS or LPS (1 µg/ml) for 3 hrs, and Nox4 (**A**) and TNF-α (**B**) mRNA expression were analyzed by qRT-PCR. (**C**) Nox4 and TNF-α protein levels were analyzed by western blotting. Densitometry analysis of Nox4 and TNF-α protein levels normalized with GAPDH in primary hepatocytes (n = 3) of each group are shown. (**D**,**E**) Primary hepatocytes isolated from Nox4 WT and Nox4 KO mice were either treated with PBS or LPS (1 µg/ml) for 3 hrs were analyzed for the cellular Nox4 (**D**) and TNF-α (**E**) protein levels by confocal microscopy. Confocal microscopic fields are shown (**D**,**E**), a representative picture of three independent experiments. * indicates statistically significant difference from the corresponding controls (p < 0.05). ^#^ indicates statistically significant difference from Nox4 WT LPS (−) (p < 0.05). Lines with p values also indicate statistically significance (p < 0.05) between the groups.

To confirm the role of Nox4 in the LPS-induced TNF-α elevation in hepatocytes, we next used primary murine hepatocytes isolated from Nox4 wild type (Nox4 WT) and Nox4 knockout (Nox4 KO) mice. LPS treatment significantly increased Nox4 mRNA in the primary hepatocytes from Nox4 wild type mice (n = 3), while Nox4 was undetectable in primary hepatocytes from Nox4 knockout mice (n = 3) (Fig. 4A). Furthermore, the LPS-stimulated increase in TNF-α mRNA was decreased in primary hepatocytes from Nox4 KO mice (n = 3) compared with hepatocytes from Nox4 wild type mice (n = 3) (Fig. 4B). Western blot and immunofluorescence analysis confirmed increased Nox4 and TNF-α protein levels upon LPS stimulation in the primary hepatocytes from Nox4 wild type mice (n = 3), while a significant decrease was observed in LPS-induced TNF-α levels in primary hepatocytes from Nox4 KO mice (n = 3) (Fig. 4C,D and E). These results suggest that Nox4 can modulate the LPS induced TNF-α upregulation in hepatocytes.

### Nox4 knockdown inhibited LPS induced ERK1/2, AP1, and NF-ĸB activation

**Figure 5:**
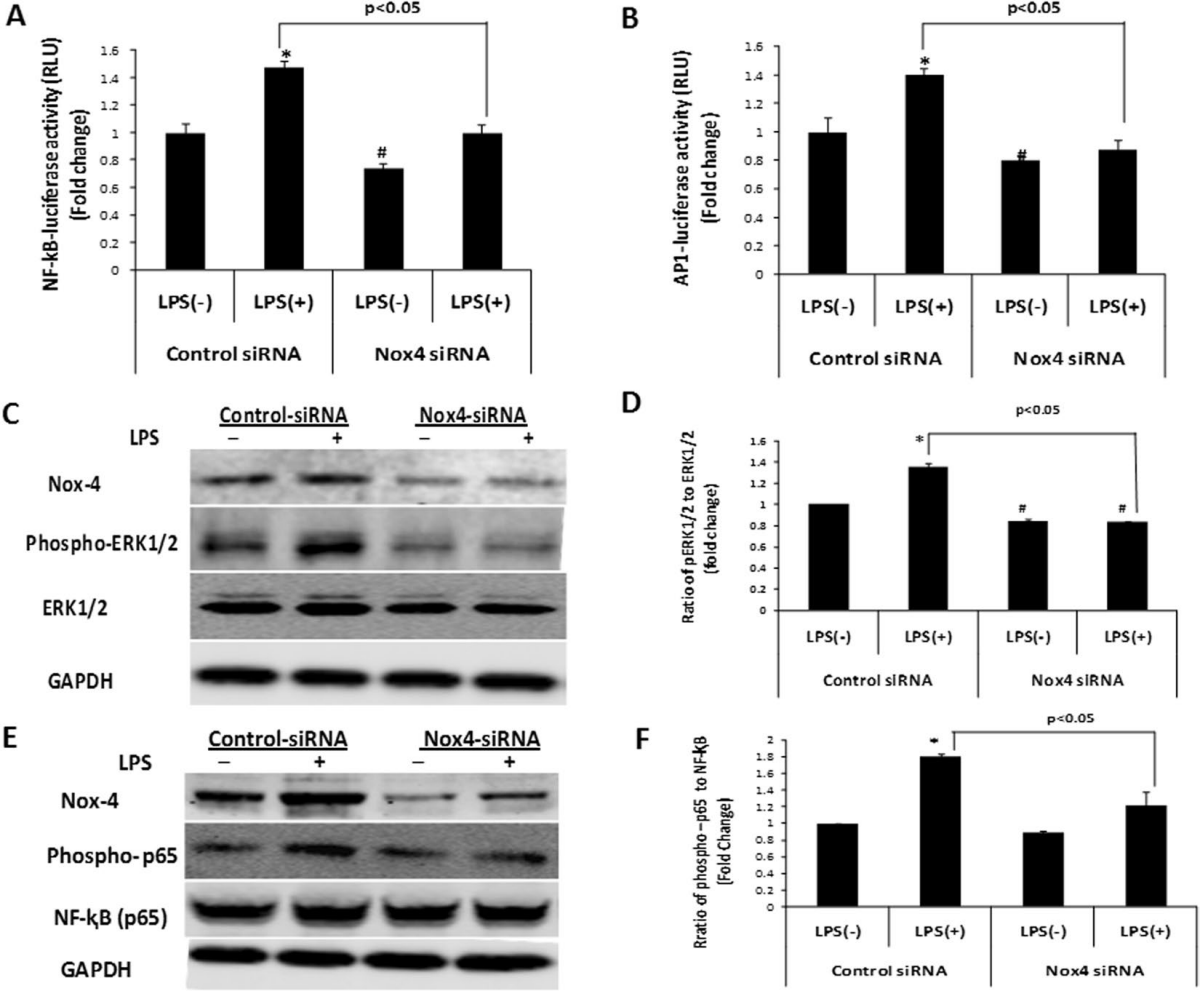
Nox4 downregulation suppressed the LPS induced NF-ĸB and ERK1/2 mediated AP1 activation. (**A**,**B**) Huh7 cells were either transfected with control or Nox4 siRNA. Then, after 24 hrs, cells were transfected with either NF-ĸB-dependent luciferase or AP1-dependent luciferase reporter construct and after 24 hrscells wereeither treated with PBS or LPS for an additional 6 hrs. Luciferase activity was determined by luciferase reporter assay as described in materials and methods. (**C**–**F**) Huh7 cells transfected with control or Nox4 siRNA for 48 hrs, and either treated with LPS or PBS for additional 6 hrs. Nox4, phospho-ERK1/2, ERK1/2, phospho-NF-ĸB p65 and NF-ĸB p65 protein levels were analyzed by western blotting. Representative data of three independent experiments (n = 3) are shown. Relative ratios of p-ERK1/2 to ERK1/2 (**D**) and p-NF-ĸB to NF-ĸB (**F**) were quantified by densitometry and normalized by the level of GAPDH. * indicates statistically significant difference from the corresponding controls (p < 0.05). ^#^ indicates statistically significant difference from control siRNA LPS (−) (p < 0.05). Lines with p values also indicate statistically significance (p < 0.05) between the groups.

Recent studies indicated that LPS-TLR4 signaling in cancer cells may promote cell survival, proliferation and epithelial-mesenchymal transition in HCC via extracellular signal-regulated kinase (ERK) and NF-ĸB pathways^35,36^. On the other hand, it has been reported that Nox4 is involved in LPS-stimulated NF-ĸB and ERK1/2- or JNK-mediated AP-1 activation in human aortic endothelial cells and human aortic smooth muscle cells, respectively^20,37^. To further determine the role of Nox4 in the LPS-TLR4 signaling pathway in hepatocytes, Huh7 cells treated with control siRNA or Nox4 specific siRNA were transfected with NF-ĸB or AP-1 dependent luciferase reporter and analyzed for luciferase activity after LPS treatment. LPS treatment resulted in marked increases in NF-ĸB and AP-1 reporter expression. In contrast, Nox4 siRNA attenuated the NF-ĸB and AP-1 reporter expression, compared to control siRNA-transfected cells, in the presence of LPS (Fig. 5A and B). LPS also increased p65 and ERK1/2 phosphorylation in Huh7 cells, whereas Nox4 knockdown significantly decreased the LPS-induced phosphorylation of ERK1/2 and p65 (Fig. 5C–F). Together, these results suggest that Nox4 modulates LPS induced NF-ĸB and ERK1/2 activation in these cells.

### Nox4 deletion altered the responses to LPS in the liver and plasma in mice

**Figure 6:**
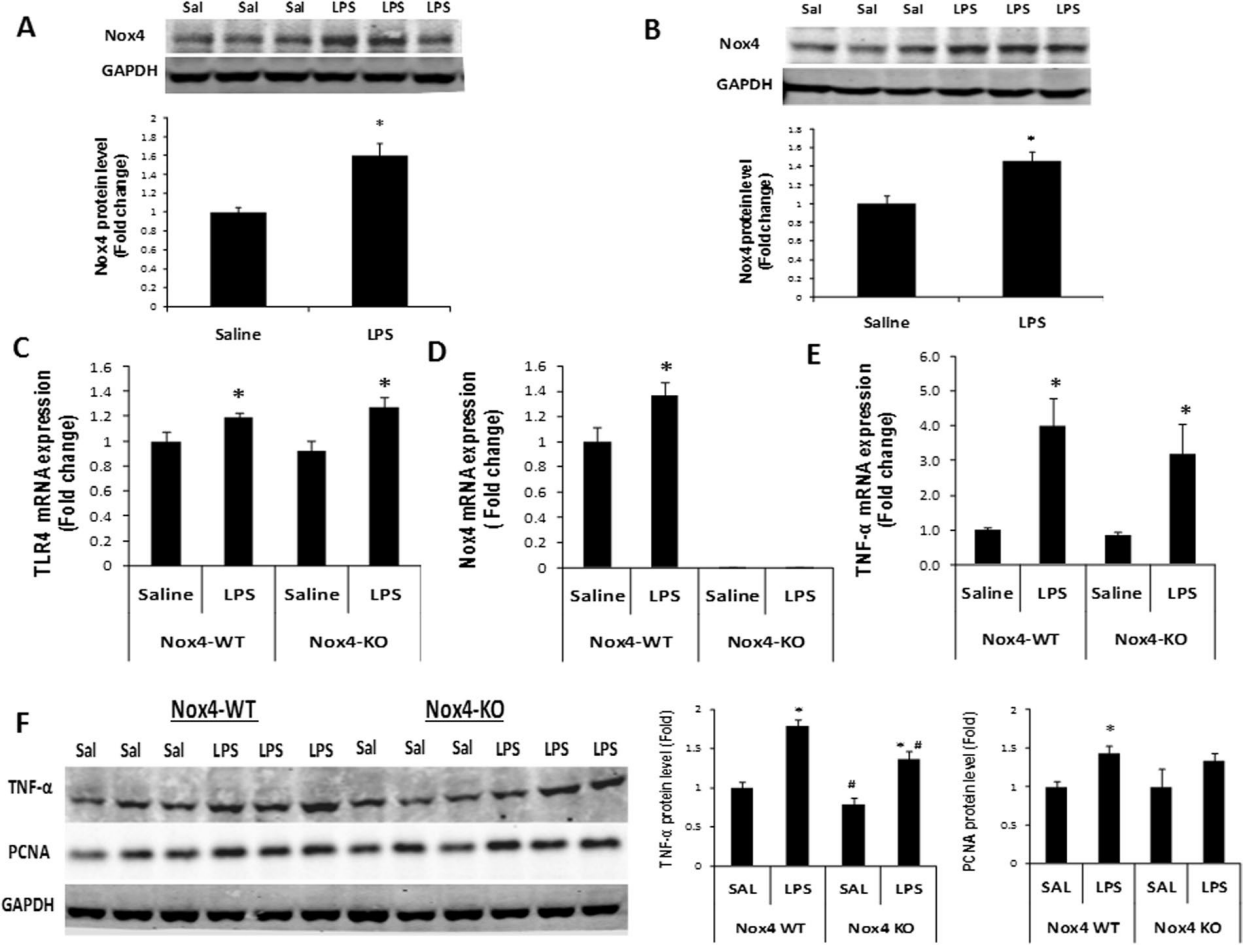
LPS increased TNF-α and PCNA levels in the liver of Nox4 WT and Nox4 KO mice after 24 hrs of stimulation. Wild type mice were injected with saline (n = 3) or LPS (n = 3) (1 mg/kg body weight) either only for 24 hrs (**A**) or up to 6 weeks (**B**) of repeated stimulation (once a week). Nox4 protein levels were determined by western blotting. Nox4 protein levels after 24 hrs (**A**) and 6 weeks (**B**) of repeated LPS treatment were quantified by densitometry and normalized with the GAPDH levels. Wild type (n = 3) and Nox4 KO (n = 3) mice were either injected with saline or LPS for 24 hrs, and TNF-α, Nox4 and PCNA levels were analyzed in the liver. (**C**–**E**) Liver samples (n = 3) of each group were analyzed for TLR4, Nox4, and TNF-α mRNA expression by qRT-PCR. (**F**) TNF-α, and PCNA protein levels were analyzed by western blotting. Densitometry analysis of TNF-αand PCNA protein levels normalized with GAPDH in liver samples (n = 3) of each group are shown. * indicates statistically significant difference from the corresponding controls (p < 0.05). ^#^ indicates statistically significant difference from Nox4 WT saline groups (p < 0.05). Lines with p values also indicate statistically significance (p < 0.05) between the groups.

We also examined whether Nox4 levels increased with LPS *in vivo*. WT mice were injected *i*.*p*. with LPS or saline alone weekly for up to six weeks and analyzed for Nox4 protein levels. Nox4 protein increased in the liver of control WT mice challenged with LPS, compared to mice injected with saline at week 1 and week 6 (n = 3 each group) (Fig. 6A,B), indicating that LPS induces Nox4 *in vitro* and *in vivo*.

**Figure 7:**
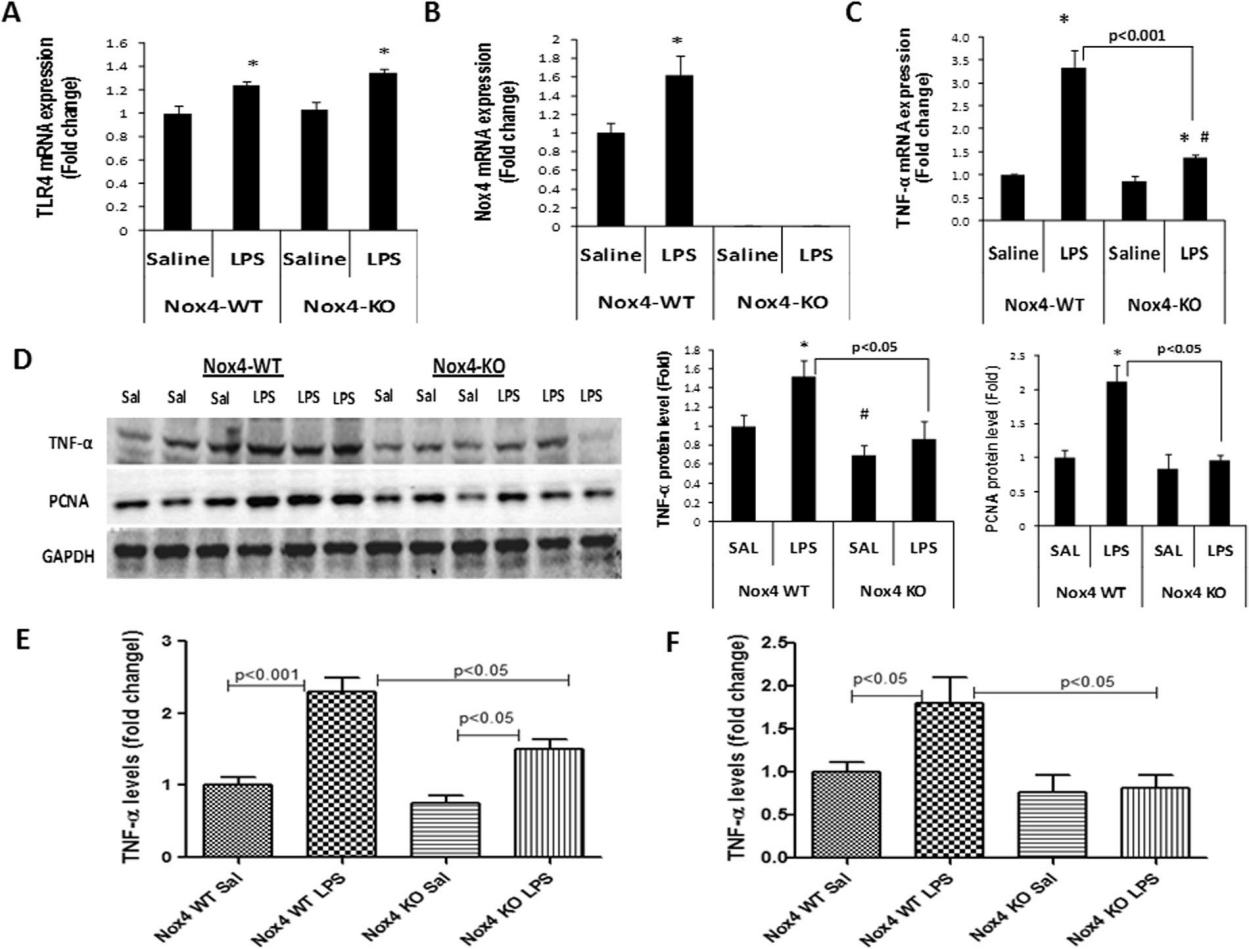
LPS induced TNF-α and PCNA levels subsided in the liver of Nox4 KO mice with six weeks of repeated LPS stimulation. Wild type and Nox4 KO mice were either injected with saline or LPS for up to 6 weeks (once a week), and TNF-α, Nox4 and PCNA levels were analyzed in the liver samples. (**A**) Liver samples (n = 3) of each group were analyzed for TLR4 mRNA expression. (**B**) Analysis of Nox4 mRNA levels in the liver samples (n = 3) of all groups. (**C**) TNF-α mRNA expression were analyzed in the liver samples of wild type (n = 7, in each group) groups and Nox4 KO (n = 5, in each group) groups. (**D**) Western blot analysis of TNF-α and PCNA protein levels in liver samples (n = 3) of all groups. (**D**) Densitometry analysis of TNF-α and PCNA protein levels normalized with GAPDH in liver samples (n = 3) of each group are shown. (**E**,**F**) TNF-α levels were analyzed in the plasma samples (n = 3) of wild type and Nox4 KO mice groups injected with saline or LPS either for 24 hrs (**E**) or up to 6 weeks (**F**) of repeated stimulation. * indicates statistically significant difference from the corresponding controls (p < 0.05). ^#^ indicates statistically significant difference from Nox4 WT saline groups (p < 0.05). Lines with p values also indicate statistically significance (p < 0.05) between the groups.

To test whether Nox4 played a role in the hepatic LPS response *in vivo* in mice, both Nox4 WT and global Nox4 KO mice were exposed to LPS or saline alone for 24 hours (week 1) and with weekly injection of LPS or saline up to 6 weeks. Then, liver samples were harvested and analyzed for Nox4, TNF-α, TLR4 and PCNA levels. The results showed that LPS elevated TLR4, Nox4 and TNF-α mRNA levels in the liver of Nox4 WT mice, compared with saline injected controls at 24 hrs after first injection during week 1 (Fig. 6C–E). Nox4 mRNA was undetectable in the Nox4 KO mice (Fig. 6D). Western blot analysis revealed increased TNF-α levels in the liver of Nox4 WT and Nox4 KO mice, compared to saline injected controls at 24 hrs after first injection during week 1 (Fig. 6F). We also detected elevated Nox activity with LPS stimulation at week 1 in the liver of WT mice, compared with saline control group, that was DPI sensitive. Nox4 KO mice exhibited decreased Nox activity compared to wild type mice and did not increase with LPS (Supplementary Fig. S6). However, the LPS-induced changes in TNF-α levels were not significantly different in Nox4 KO mice compared with Nox4 WT mice at this time point (week 1). In contrast, after 6 weeks of repeated LPS stimulation, the LPS-associated elevations in TNF-α levels were significantly attenuated in the liver of Nox4 KO mice compared with Nox4 WT liver samples (Fig. 7C,D). On the other hand, TLR4 mRNA increased to similar levels in the two animal groups (Fig. 7A). LPS increased Nox4 levels in the liver of WT mice at week 6 but no Nox4 mRNA was detected in Nox4 KO mice group (Fig. 7B).

Furthermore, the effect of *nox4* deletion on hepatic PCNA content was evaluated in the WT and global Nox4 KO mice at week 1 and 6 with repeated LPS stimulation. Mice with one as well as six weeks of repeated LPS stimulation showed marked increase in PCNA protein, compared to saline controls (Figs 6D and 7D). The LPS-stimulated changes in PCNA protein level was not significantly different in Nox4 KO mice, compared with Nox4 WT mice at week 1 (Fig. 6D). However, at week 6, a significant reduction in the LPS-stimulated PCNA elevation was notable in the Nox4 KO mouse liver, compared with WT liver (Fig. 7D). Taken together, these data suggest that Nox4 affected hepatic LPS responses upon repeated LPS stimulation *in vivo* in mice.

### TNF-α plasma levels declined in Nox4 KO mice in response to LPS

We examined next the effect of global deletion of the *nox4* gene on the plasma levels of TNF-α in responses to LPS. TNF-α plasma levels were decreased in Nox4 KO mice compared with Nox4 WT upon LPS stimulation. The TNF-α plasma levels declined further with six weeks of repeated LPS stimulation than with one injection at week 1 in Nox4 KO mice compared with Nox4 WT mice (Fig. 7E and F). These results suggest that Nox4 may play an important role in the LPS-induced TLR4-signaling pathways.

## Discussion

Chronic liver inflammation is an important contributor to hepatocellular carcinomas (HCCs), the third leading causes of cancer-related death worldwide^38^. Chronic inflammation is frequently associated with functional disruption of gut barrier and translocation of intestinal bacteria and bacterial PAMPs^3–7^. Activation of Toll-like receptors (TLRs), specifically TLR4 signaling in response to microbial lipopolysaccharide (LPS-TLR4), has emerged as a central component of the liver’s inflammatory response^4,39,40^. LPS-induced TLR4 signaling has been shown to promote hepatocarcinogenesis^13^ and hepatic fibrosis^41^. LPS increases the production of NADPH oxidase (Nox) derived reactive oxygen species through TLR4, which thereby activates the downstream signaling pathways^15–18^. Accumulating data demonstrate that Nox4 mediates LPS-TLR4 signaling (TLR4-Nox4-NF-ĸB/AP1/p38) through ROS generation in human HEK293T cells, U937 monocytic cells, human aortic endothelial cells, human renal mesanglial cells (HMRCs) and human aortic smooth muscle cells (HASMC)^9–21,37^. However, whether and how Nox4 mediates LPS-TLR4 signaling in the liver was unclear.

In the present study, we explored the contribution of Nox4 in hepatic responses induced by LPS. We found that LPS increased TNF-α and Nox4 expression in the HCC cell line, Huh7. The Nox-specific increase in ROS levels were observed with LPS. This effect was mediated by LPS-TLR4 signaling at the transcriptional level. In agreement with previous studies^4,13,14^, upregulation in the proliferation marker, PCNA, was observed along with TLR4 expression, which indicates that LPS promotes HCC proliferation. Binding of LPS to TLR4 activates both the myeloid differentiation primary-response gene88 (MyD88)-dependent and TIR-domain-containing adaptor protein inducing IFNβ (TRIF)-mediated (MyD88-independent) pathways^9,10,42^. In our study, MyD88 knockdown decreased the LPS-induced TNF-α and Nox4 elevation in Huh7 cells. This suggests that the LPS-TLR4 signaling pathway in hepatocytes is MyD88 dependent.

Our data further show that LPS-induced TNF-α and PCNA expression is mediated by Nox4 in an ROS-dependent manner. Nox4 downregulation also attenuates the proliferation of Huh7 hepatoma cells upon LPS stimulation. Activation of NF-ĸB has been strongly associated with liver inflammation and HCC through the induction of inflammatory cytokines and inhibition of apoptotic genes^43,44^. The ERK1/2 signaling is associated with cell survival and proliferation in a stimulus type and duration dependent manner^45^, whereas suppression of ERK1/2 activation can inhibit the growth of human hepatocellular carcinoma^46^. Park *et al*. have shown that NOX4 is involved in LPS induced ROS generation and NF-ĸB activation in HEK293T cells and human aortic endothelial cells^18,20^. While Devang *et al*. have demonstrated that LPS induces both NF-ĸB and ERK1/2- or JNK-mediated AP-1 activation in human aortic smooth muscle cells in a Nox4 dependent manner^37^. In the present study, we found that LPS activates both NF-ĸB and ERK1/2 signaling pathways in human HCC cells; however, Nox4 silencing suppressed the LPS-induced NF-ĸB and ERK1/2-mediated AP-1 activation. We have not determined whether AP-1 is activated through JNK pathway. The results of previous studies together with the present data indicate that Nox4 mediates the LPS-TLR4 signaling in human hepatoma cells.

In this study, we found that LPS increased the Nox4 levels in primary hepatocytes isolated from the liver of mice. However, LPS induced TNF-α levels were subsided in the primary hepatocytes of Nox4 knockout mice. Further, LPS stimulation up to six weeks in the *in vivo* mice model increased TNF-α and PCNA levels in a Nox4-dependent manner. The hepatic TNF-α and PCNA responses were attenuated more in the six weeks of repeated LPS stimulation than 24 hrs (week 1) in the liver of Nox4 knockout mice compared to the wild type mice, while the wild type mice continued to show elevated levels of TNF-α and PCNA with LPS stimulation. TNF-α plasma levels also declined in Nox4-deficient mice compared to wild type in response to LPS. Our results show that the effect of Nox4 deletion on *in vivo* hepatic and plasma responses to LPS stimulation was time-dependent.

Recent studies have shown that TLR4 and intestinal microbiota are involved in carcinogen induced hepatocarcinogenesis, and sterilization of gut decreased the incidence of HCC^4,13^. Dapito *et al*. demonstrated that TLR4 and intestinal microbiota were required for HCC promotion but not for HCC initiation in chronically injured liver^13^. Our results indicate that LPS increased the proliferation of HCC cells in a Nox4-dependent manner. We found that LPS stimulation also increased the proliferation of liver hepatocytes in mice, whereas Nox4 knockout mice showed a decrease in proliferation as observed by PCNA level in a time dependent manner. These data show that LPS promotes hepatocyte proliferation in mice.

In summary, the present study provides evidence that Nox4 mediates LPS-TLR4 signaling in human hepatoma cells and murine hepatocytes *in vitro* through the TLR4-Nox4-NF-κB and TLR4-Nox4-AP-1 signaling pathways. Nox4 may also play a role in hepatic and plasma responses to repeated LPS exposure *in vivo*.

## Electronic supplementary material

Supplementary figures
