# Supplementary material for: NADPH oxidase 4 modulates hepatic responses to lipopolysaccharide mediated by Toll-like receptor-4
Source: Sci Rep. 2017 Oct 30;7:14346. doi: 10.1038/s41598-017-14574-8 (PMC5662726; doi:10.1038/s41598-017-14574-8)
Supplement: Supplementary file 1 — Supplementary figures [file 41598_2017_14574_MOESM1_ESM.pdf]

Supplementary data for:

"NADPH oxidase 4 modulates hepatic responses to lipopolysaccharide mediated by Toll-like receptor-4"

by Anand Singh, Bhargav Koduru, Cameron Carlisle, Hasina Akhter, Rui-Ming Liu, Katrin Schroder, Ralf P. Brandes, David M. Ojcius

Supplementary Fig. S1

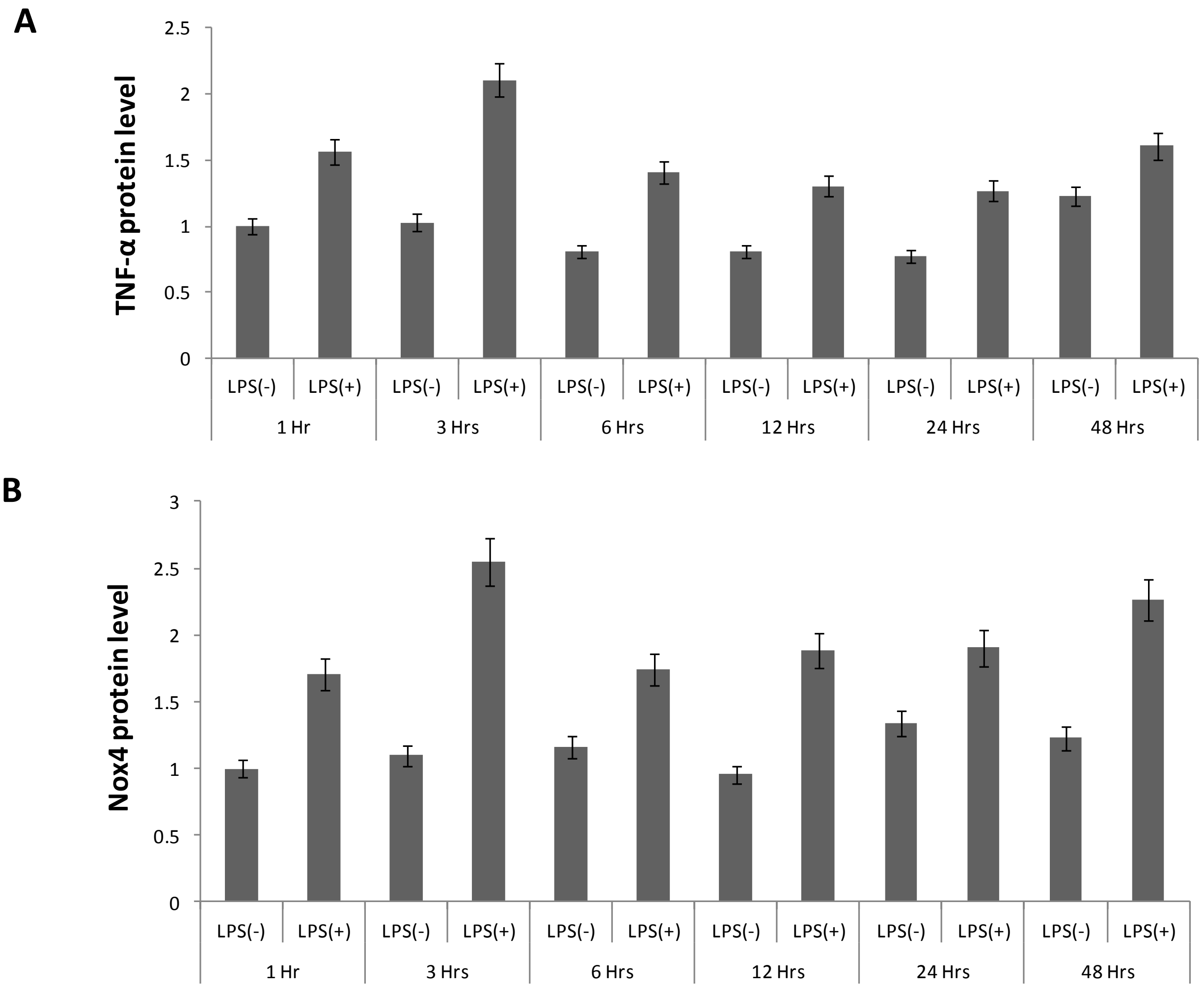

Supplementary Figure S1. LPS increased TNF-α and Nox4 protein levels in Huh7 cells. Huh7 cells were treated with LPS (1µg/ml) for up to 48 hrs, and analyzed for TNF-α and Nox4 protein levels by western blot. TNF-α (A) and Nox4 (B) protein levels were quantified by densitometry and normalized by GAPDH protein levels at time points indicated (n=2).

Supplementary Fig. S2

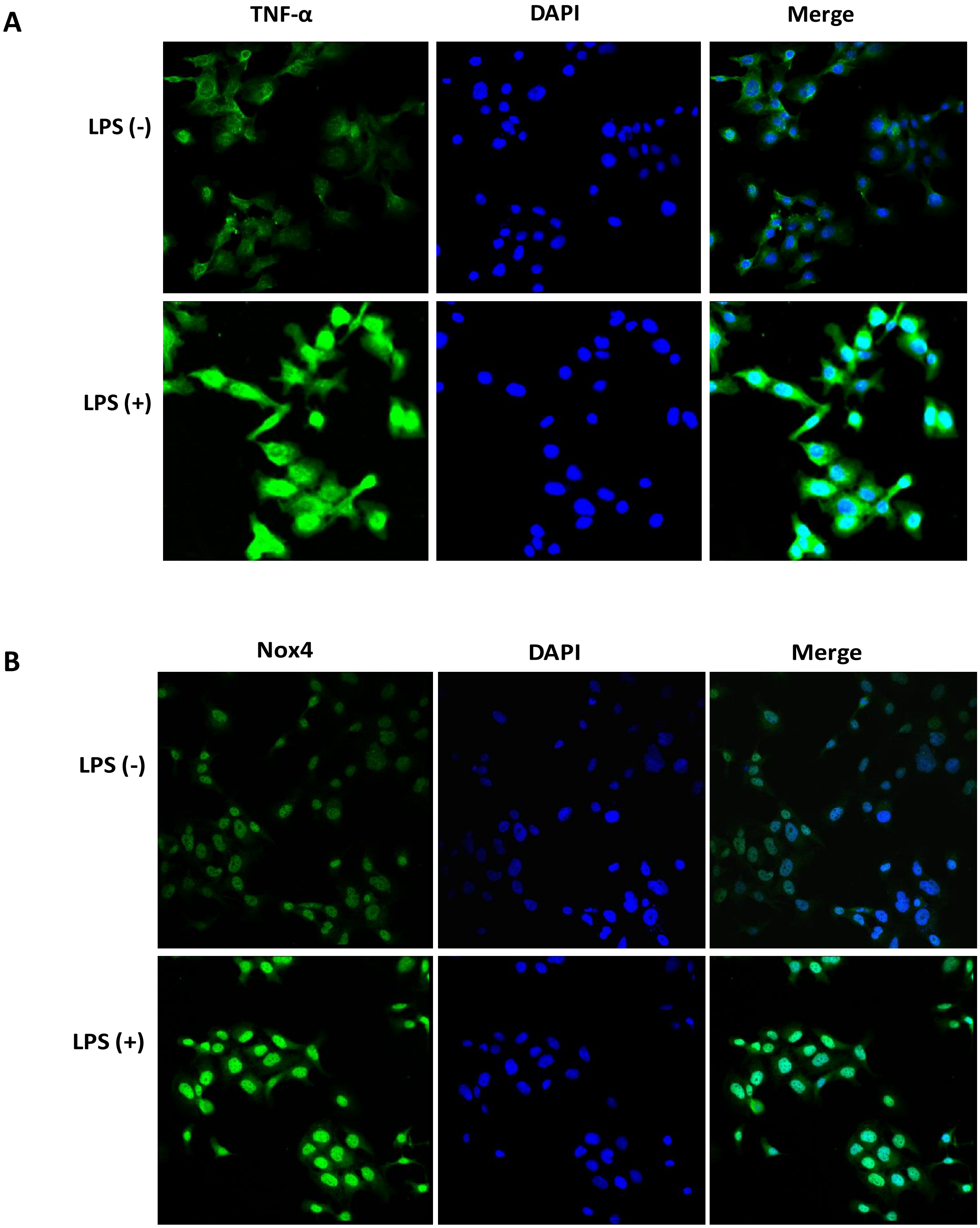

Supplementary Figure S2. Analysis of TNF-α and Nox4 protein levels in Huh7 cells treated with LPS by Immunocytochemistry staining. Huh7 cells either untreated or treated with LPS for

3 hrs and TNF-α (A) and Nox4 (B) protein levels were analyzed by confocal microscopy. Confocal microscopic fields are shown here, a representative picture of three independent experiments.

Supplementary Fig. S3

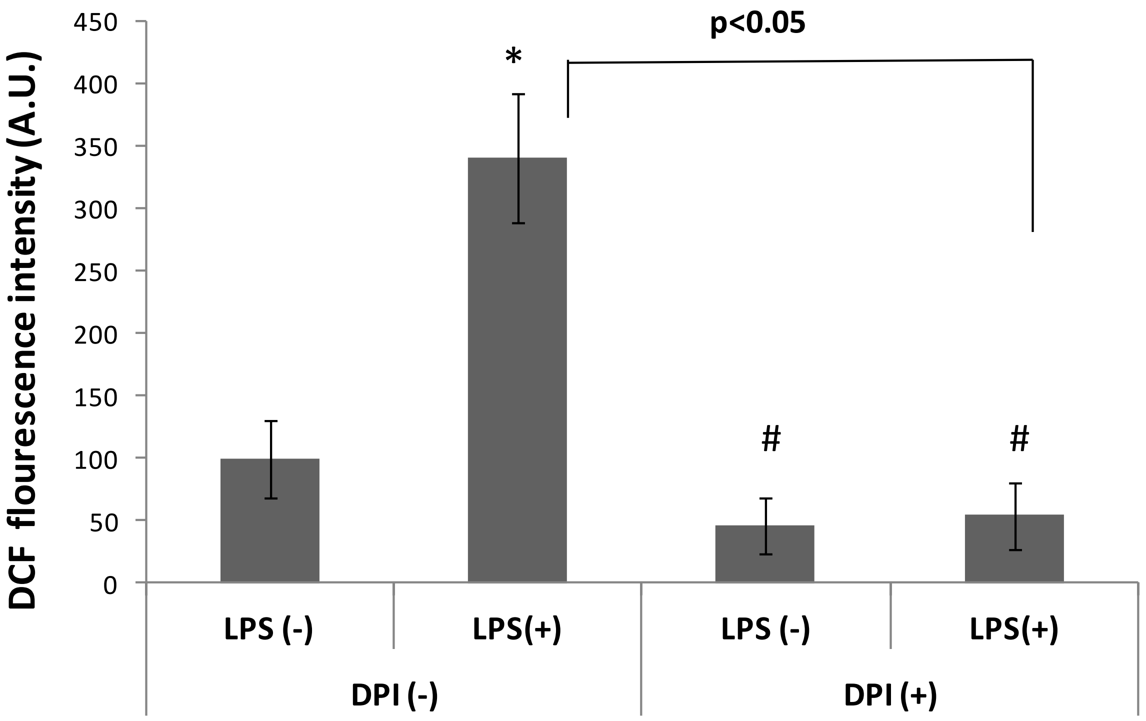

Supplementary Figure S3. LPS mediated generation of H_2O_2. Huh7 cells were either untreated or pretreated with DPI (10µM) for 30 minutes before 3 hrs of LPS (1µg/ml) stimulation. Then, cells were labeled with CM-H2DCFDA (5 µM) for 15 min and intracellular generation of $H_2O_2$was monitored by confocal microscopic analysis of DCF fluorescence. Fluorescence intensity relative to unstimulated (LPS-) cells is shown. Data are mean±SE from three independent experiments. * indicates statistically significant difference from the corresponding controls ($p<0.05$). [#] indicates statistically significant difference from control LPS (-) ($p<0.05$). Lines with p values also indicate statistically significance ($p< 0.05$) between the groups.

Supplementary Fig. S4

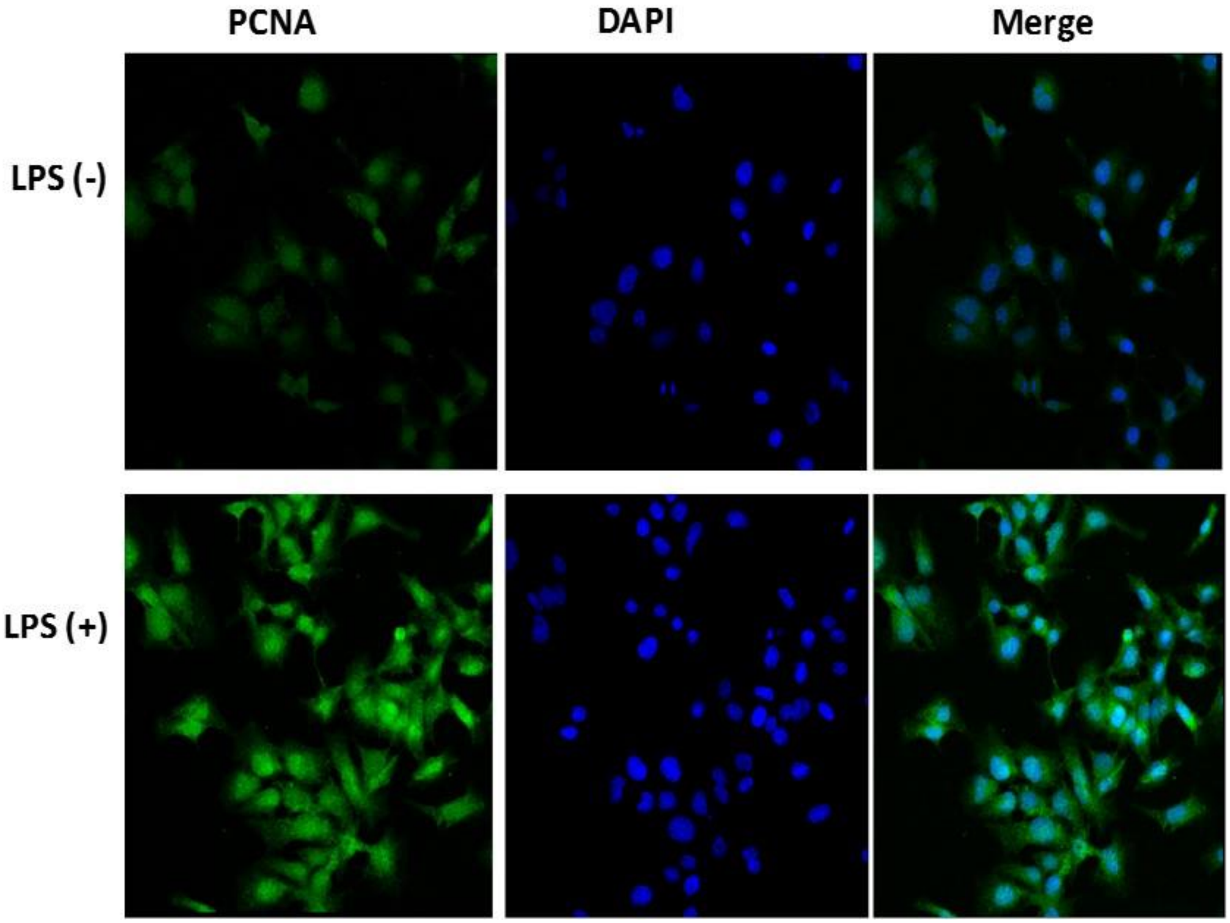

Supplementary Figure S4. LPS increased PCNA protein levels in Huh7 cells. Huh7 cells either untreated or treated with LPS for 3 hrs and PCNA protein signal were analyzed by confocal microscopy. Confocal microscopic fields are shown here, a representative picture of three independent experiments.

Supplementary Fig. S5

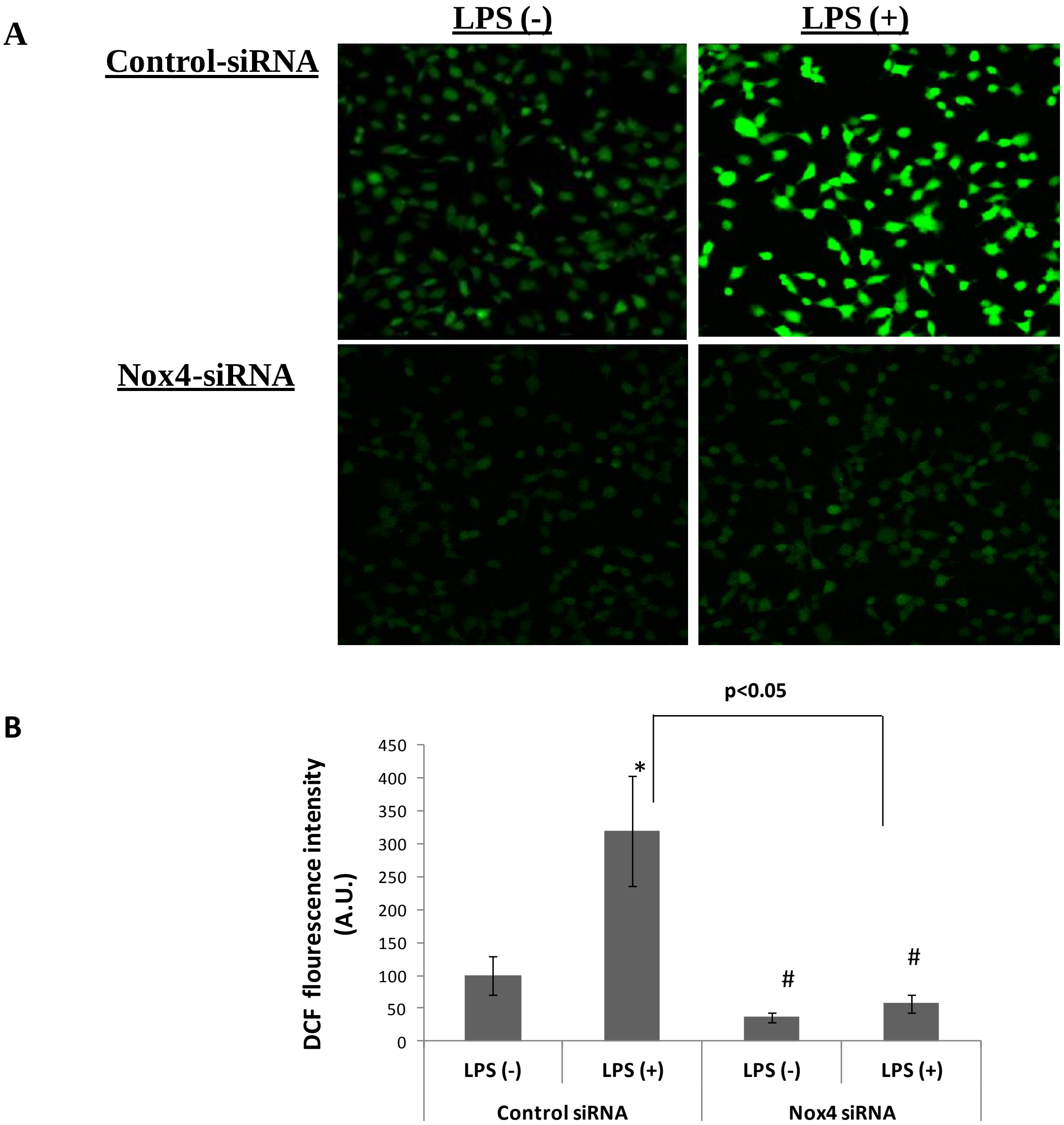

Supplementary Figure S5. Effect of Nox4 silencing on LPS-induced H_2O_2 generation. Huh7 cells transfected with control or Nox4 siRNA for 48 hrs followed by 3 hrs LPS stimulation. The generation of intracellular H_2O_2 was monitored by confocal microscopic analysis of DCF fluorescence in the absence or presence of LPS. Representative microscopic fields are shown in (A) and the fluorescence intensity relative to unstimulated (LPS -) cells is shown in (B). Data in B are mean± SE of three independent experiments. * indicates statistically significant difference

from the corresponding controls ($p<0.05$). # indicates statistically significant difference from control LPS (-) ($p<0.05$). Lines with p values also indicate statistically significance ($p < 0.05$) between the groups.

Supplementary Fig. S6

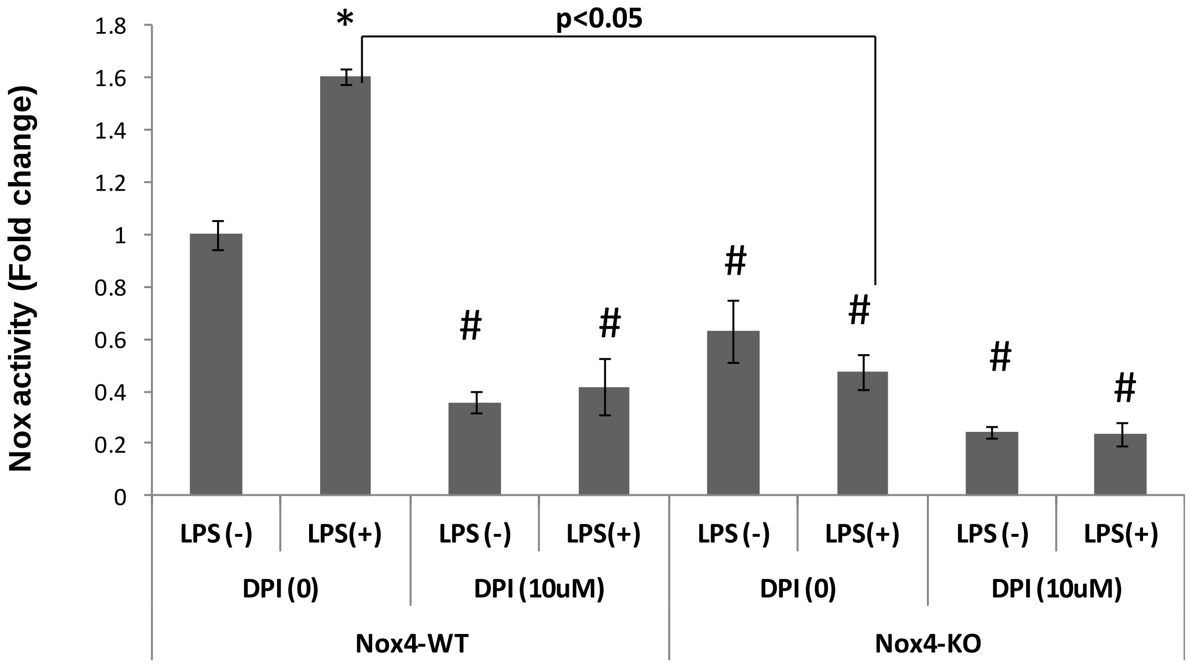

Supplementary Figure S6. Activity of Nox enzyme decreases in the liver of Nox4 KO mice injected with LPS. Wild type and Nox4 KO mice were injected with saline or LPS for 24 hrs (week 1), and liver samples (n=3) of each group were analyzed for Nox activity. Mice liver samples of each group were sonicated in intracellular-like buffer, and SOD inhibited reduction of cytochrome c was determined in the presence and absence of DPI, as described in materials and methods. *****indicates statistically significant difference from the corresponding controls ($p<0.05$). # indicates statistically significant difference from Nox4 WT saline groups ($p<0.05$). Lines with p values also indicate statistically significance ($p< 0.05$) between the groups.

Supplementary table I. List of primers used for qRT-PCR.

Set	Gene	Direction	Sequence (5' to 3')
1	Human Nox4	Sense	TCACAAGGTTCCAAGCAG
		Antisense	ACTGAGAAGTTGAGGGCATTC
2	Human TNFα	Sense	CCATGTTGTAGCAAACCCTCAA
		Antisense	GCTGGTTATCTCTCAGCTCCA
3	Human PCNA	Sense	GAGGAGGAAGCTGTTACCATAGA
		Antisense	TGAGTGTCACCGTTGAAGAGA
4	Human MyD88	Sense	TCGAGACCTCAAGGGTAGA
		Antisense	GCGCTTCCTCTTTCTCCT
5	Human TLR4	Sense	GAACCTGGACCTGAGCTTTAAT
		Antisense	GTCTGGATTTCACACCTGGATAA
6	Human GAPDH	Sense	GGTGGTCTCCTCTGACTTCAA
		Antisense	GTTGCTGTAGCCAAATTCGTT
7	Mouse Nox4	Sense	GGATCACAGAAGGTCCCTAGCAG
		Antisense	GCGGCTACATGCACACCTGAGAA
8	Mouse TNFα	Sense	AGCAAACCACCAAGTGGAGGA
		Antisense	GCTGGCACCACTAGTTGGTTGT
9	Mouse TLR4	Sense	GTCCCTGATGACATTCCTTCTTC
		Antisense	CCAGCCACTGAAGTTCTGAAA
10	Mouse GAPDH	Sense	CAATGTGTCCGTCGTGGATCT
		Antisense	TTGAAGTCGCAGGAGACAACC
